# Lymphoepithelioma-like carcinoma of the distal urethra: a case report and management strategies

**DOI:** 10.1093/jscr/rjaf785

**Published:** 2025-10-03

**Authors:** Ramiro Fonseca, José Ignacio Perez Reggeti, Cristina Ferreiro Pareja, Gabriela Paganini, Marcial Berrios-Quinteros, Gilberto Eduardo Chechile, Nahuel Paesano

**Affiliations:** Department of Urology, Instituto Médico Tecnológico, Carrer Escorial 171, Barcelona 08024, Spain; Department of Urology, Hospital Universitario Bellvitge, Carrer de la Feixa Llarga s/n, Barcelona 08907, Spain; Department of Urology, Hospital Universitario Bellvitge, Carrer de la Feixa Llarga s/n, Barcelona 08907, Spain; Department of Urology, Instituto Médico Tecnológico, Carrer Escorial 171, Barcelona 08024, Spain; Department of Urology, Instituto Médico Tecnológico, Carrer Escorial 171, Barcelona 08024, Spain; Department of Urology, Instituto Médico Tecnológico, Carrer Escorial 171, Barcelona 08024, Spain; Department of Urology, Instituto Médico Tecnológico, Carrer Escorial 171, Barcelona 08024, Spain

**Keywords:** lymphoepithelioma-like carcinoma of the urethra, rare genitourinary tumors, urethral cancer, oncology urology, HPV-related carcinoma

## Abstract

Lymphoepithelioma-like carcinoma (LELC) of the urethra is an exceptionally rare and aggressive neoplasm, with limited evidence available to inform optimal management. We present the case of a 63-year-old male diagnosed with high-grade urethral LELC, who presented with locally advanced disease. The patient underwent partial penectomy with bilateral inguinal lymphadenectomy, followed by systemic chemotherapy and immunotherapy. This case underscores the importance of a multidisciplinary approach combining surgical resection with systemic therapy in the management of aggressive urethral malignancies. Given the paucity of literature on urethral LELC, additional case reports are critical to enhance our understanding and to develop evidence-based treatment strategies for this rare entity.

## Introduction

Primary urethral carcinoma (PUC) is rare, accounting for less than 1% of genitourinary malignancies. The most common histological subtype is urothelial carcinoma, particularly in the proximal urethra, comprising approximately 75% of cases. PUC shows a male predominance (male/female ratio 2.9:1) and typically affects individuals over 75 years of age [1].

PUC has been associated with several predisposing factors, including urethral strictures, chronic irritation due to intermittent catheterization or urethroplasty, external beam radiation therapy, radioactive seed implantation, chronic urethritis, sexually transmitted infections, especially Human Papillomavirus (HPV)-16, and lichen sclerosus [2].

Lymphoepithelioma-like carcinoma (LELC) is a rare histological variant of PUC, representing less than 1% of cases. It mimics the morphology of nasopharyngeal lymphoepithelioma. While LELC has been described in the urinary bladder and renal pelvis, distal urethral cases are scarcely reported [3]. This case report aims to expand the limited knowledge on this variant by presenting the clinical features, diagnosis, treatment, and outcomes in a male patient with distal urethral LELC.

## Case presentation

**Figure 1:**
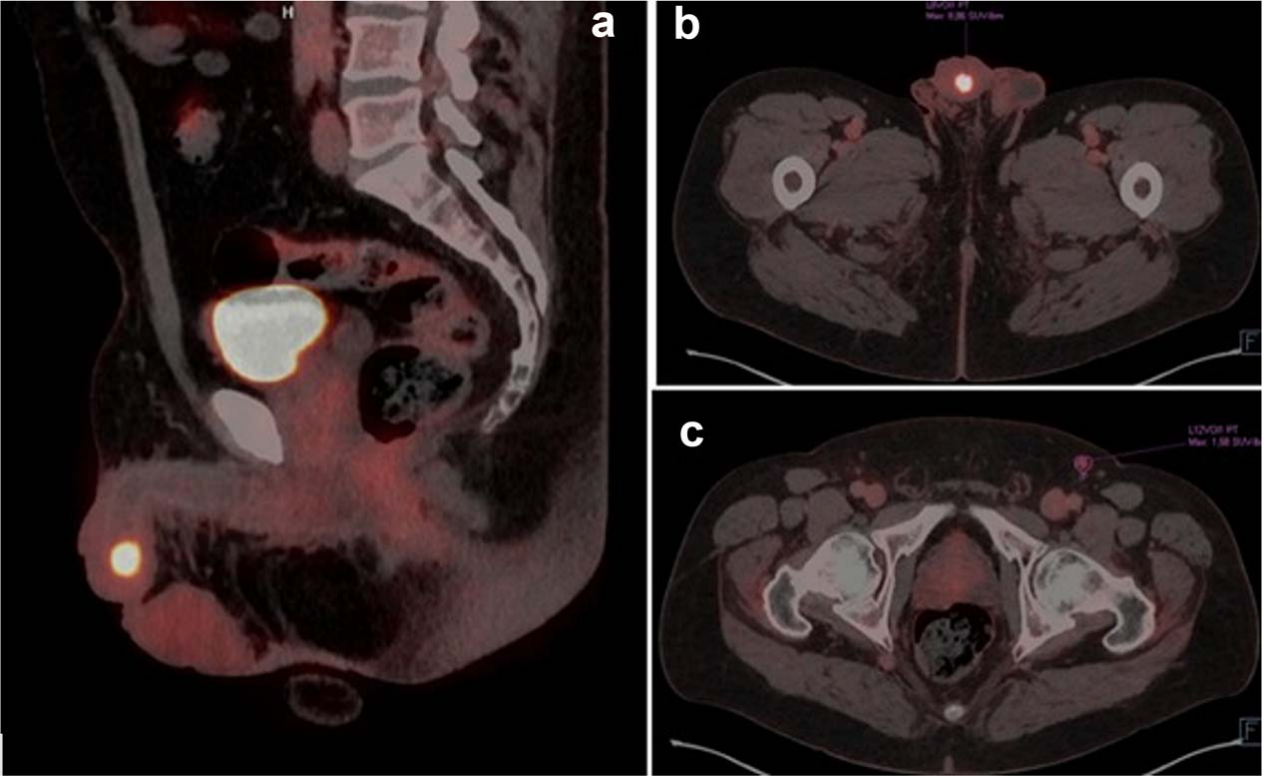
(a and b) PET-TC FDG-18 showing hypermetabolic lesion in glans. (c) Left inguinal lymph node positive in PET-TC.

A 63-year-old male presented with a three-year history of reduced urinary flow and dysuria, developing urethral bleeding in the last 8 months prior to consultation. Two weeks before seeking care at our institution, he underwent holmium laser transurethral resection of a hardened papillomatous urethral lesion located in the navicular fossa. Pathological analysis revealed invasion by pure LELC of the urethra with involvement of the lamina propria (pT1). Advanced imaging studies, including penile MRI and FDG-18 PET-CT, demonstrated involvement of the glans and corpus spongiosum by the lesion (Fig. 1a–c).

The urethrocystoscopy did not reveal any other suspicious lesions in the rest of the urethra or in the bladder. Additional testing for HPV was negative.

**Figure 2:**
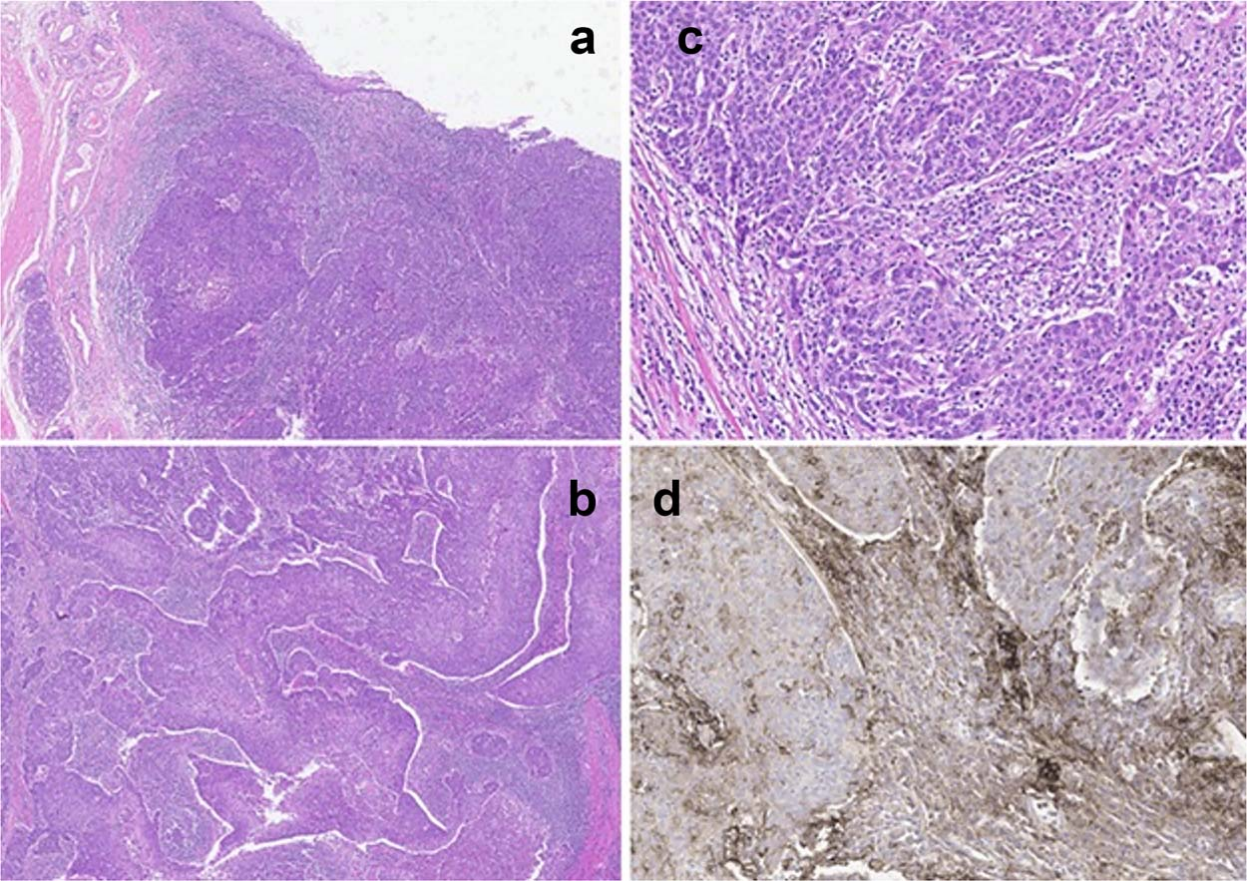
(a) Panoramic view of tumor located in the distal urethra with infiltration of connective tissue without extension to the muscular layer or cavernous body. (b) Tumor with syncytial pattern associated with marked mixed inflammatory infiltrate that partially masks the tumor cells. (c) Tumor cells with large and pleomorphic nuclei, prominent nucleoli, poorly defined borders, and frequent mitosis. (d) Immunohistochemical staining for PDL-1 shows intense positivity in the inflammatory component and in the tumor.

The patient underwent a partial penectomy. The conclusive diagnosis identified a 3 × 2 cm mass in distal urethra high-grade pure lymphoepithelioma-like tumor originating from the urethra. Immunohistochemical testing showed positive results for the p16, p63, and PD-1 markers, and negative results for Uroplakin-III (Fig. 2a–d).

Two weeks postoperatively, FDG PET-CT detected a hypermetabolic left inguinal lymph node (SUVmax 10.77). Bilateral inguinal lymphadenectomy revealed one metastatic node (1/17) on the left side and none on the right (0/15). The postoperative course was complicated by a lymphatic abscess requiring drainage.

The oncology board recommended adjuvant chemotherapy with four cycles of cisplatin and gemcitabine, which were well tolerated. Three months later, follow-up FDG PET-CT revealed a right inguinopubic recurrence outside the prior surgical field. Needle biopsy confirmed metastasis, and subsequent lymphadenectomy removed eight nodes, with one positive for disease.

Given disease progression, PD-L1 testing was performed and pembrolizumab 200 mg every 21 days was initiated, administered for 12 months. Following nodal involvement and recurrence after chemotherapy, the decision to perform a robotic-assisted pelvic lymphadenectomy was made by a multidisciplinary tumor board; 23 nodes were resected, all of which were negative for metastasis.

The patient remains under close follow-up. Two years after diagnosis, both PET-CT and urethrocystoscopy showed no evidence of disease recurrence.

## Discussion

LELC is an uncommon variant of poorly differentiated carcinoma with morphological resemblance to nasopharyngeal lymphoepithelioma, which is often EBV-associated. However, LELC of the urinary tract generally lacks EBV or HPV associations [4].

Histologically, it is composed of large undifferentiated epithelial cells in syncytial nests with prominent nucleoli and a dense lymphoid stroma. Immunohistochemistry is essential to distinguish LELC from lymphoma. Based on the classification of Amin, LELC is categorized as pure (100%), predominant (>50%), or focal (<50%) based on the extent of LELC component. Prognosis varies accordingly pure and predominant forms have better outcomes than focal forms [3].

LELC typically expresses cytokeratin, p16, and p63, and lacks p53 and HPV-related markers. CD30 may be seen in nasopharyngeal tumors but is negative in urinary LELC. A 2007 Johns Hopkins study confirmed EBV negativity in 26 out of 28 urinary LELCs [5].

Williamson *et al*. reported that 50% of bladder LELCs were associated with urothelial carcinoma in situ, though EBV and HPV tests were negative. This supports a potential pathogenesis similar to that of high-grade nonpapillary urothelial carcinomas [5].

Prognosis depends in part on tumor location; distal urethral carcinomas have better outcomes and allow for penile-sparing strategies, whereas positive proximal margins increase the risk of recurrence [6]. In bladder LELC, transurethral resection alone is inadequate, and radical surgery ensures better disease control. PET-FDG is useful for identifying early local or distant recurrence, with high specificity despite limited sensitivity [7].

Chemotherapy (e.g. cisplatin-based regimens) has shown efficacy, especially in pure/predominant forms. Radiotherapy may be an option for unfit patients. Survival rates exceed 90% in pure/predominant forms, while they approach 0% in focal LELC [8].

Although data on lymphadenectomy in urethral LELC are scarce, nodal drainage patterns suggest inguinal and pelvic dissection should be considered when the lesion extends proximally [9].

PD-L1 expression in LELC highlights the potential role of immunotherapy. Reports support the use of agents like Avelumab in advanced penile cancer, offering a possible therapeutic alternative for aggressive urethral LELC [10].

In our patient, the tumor was classified as a pure LELC, which is associated with a more favorable prognosis compared to focal forms. No conventional risk factors, such as HPV infection, urethral stricture, chronic catheterization, or prior radiotherapy, were identified. Despite achieving an R0 resection with partial penectomy, the patient developed a right inguinal recurrence shortly after adjuvant GC chemotherapy. This underscores the aggressive potential of urethral LELC even in its pure forms. The decision to perform inguinal and subsequently pelvic lymphadenectomy was consistent with the lymphatic drainage patterns described for distal urethral tumors and with the recommendations to extend surgical staging in cases of nodal involvement. Moreover, the strong PD-L1 expression observed in this case justified the initiation of pembrolizumab, administered for 12 months, which has shown promising results in other genitourinary LELCs and in advanced penile carcinoma. After multimodal management combining surgery, cisplatin-based chemotherapy, and immunotherapy, our patient remains disease-free at two years, highlighting the value of an individualized multidisciplinary approach in the absence of standardized guidelines.

## Conclusion

LELC of the distal urethra is a rare but aggressive neoplasm requiring multimodal management. Accurate diagnosis, histological classification, and staging are crucial. Surgical resection, lymphadenectomy, and systemic therapies, potentially including immunotherapy, may optimize outcomes. Accumulation of more case reports is essential to guide treatment protocols in this rare entity.
